# Characterizing memory T helper cells in patients with psoriasis, subclinical, or early psoriatic arthritis using a machine learning algorithm

**DOI:** 10.1186/s13075-021-02714-5

**Published:** 2022-01-19

**Authors:** Hannah den Braanker, Wida Razawy, Kim Wervers, Anne-Marie C. Mus, Nadine Davelaar, Marc R. Kok, Erik Lubberts

**Affiliations:** 1 Department of Rheumatology, Erasmus MC, University Medical Center, Rotterdam, The Netherlands; 2 Department of Rheumatology and Clinical Immunology, Maasstad Hospital, Rotterdam, The Netherlands; 3 Department of Immunology, Erasmus MC, University Medical Center, Rotterdam, The Netherlands

**Keywords:** Psoriatic arthritis, T cells, Machine learning algorithms

## Abstract

**Background:**

Psoriasis patients developing psoriatic arthritis (PsA) are thought to go through different phases. Understanding the underlying events in these phases is crucial to diagnose PsA early. Here, we have characterized the circulating memory T helper (Th) cells in psoriasis patients with or without arthralgia, psoriasis patients who developed PsA during follow-up (subclinical PsA), early PsA patients and healthy controls to elucidate their role in PsA development.

**Methods:**

We used peripheral blood mononuclear cells of sex and age-matched psoriasis patients included in Rotterdam Joint Skin study (*n*=22), early PsA patients included in Dutch South West Early Psoriatic Arthritis Cohort (DEPAR) (*n*=23) and healthy controls (HC; *n*=17). We profiled memory Th cell subsets with flow cytometry and used the machine learning algorithm FlowSOM to interpret the data.

**Results:**

Three of the 22 psoriasis patients developed PsA during 2-year follow-up. FlowSOM identified 12 clusters of memory Th cells, including Th1, Th2, Th17/22, and Th17.1 cells. All psoriasis and PsA patients had higher numbers of Th17/22 than healthy controls. Psoriasis patients without arthralgia had lower numbers of CCR6^-^CCR4^+^CXCR3^+^ memory Th cells and higher numbers of CCR6^+^CCR4^-^CXCR3^-^memory Th cells compared to HC. PsA patients had higher numbers of Th2 cells and CCR6^+^CCR4^+^CXCR3^-^ cells, but lower numbers of CCR6^+^CCR4^+^CXCR3^+^ memory Th cells compared to HC. The number of CCR6^+^ Th17.1 cells negatively correlated with tender joint counts and the number of CCR6^+^ Th17 cells positively correlated with skin disease severity.

**Conclusions:**

Unsupervised clustering analysis revealed differences in circulating memory Th cells between psoriasis and PsA patients compared to HC; however, no specific subset was identified characterizing subclinical PsA patients.

**Supplementary Information:**

The online version contains supplementary material available at 10.1186/s13075-021-02714-5.

## Introduction

Psoriatic arthritis (PsA) is a multifaceted chronic rheumatic disease characterized by psoriatic skin lesions, arthritis, enthesitis, dactylitis, and/or axial disease. Approximately 30% of psoriasis patients develop PsA [1]. Psoriasis patients developing PsA are thought to go through different phases starting with aberrant activation of the interleukin (IL) 17–IL-23 axis, followed by a silent inflammatory phase (only visible by imaging), passing on to a transition phase characterized by arthralgia and fatigue and ending with clinically evident PsA [2].

Whether all psoriasis patients developing PsA undergo all of these phases is unclear, and the exact triggers and events underlying the transition from one phase to another are also not identified yet. However, it is clear that the IL-17-IL-23 axis plays a crucial role in this process. Although different cell types such as CD8^+^ T cells, type 3 innate lymphoid cells, ɣɗ T cells, and memory CD4^+^ T cells can produce IL-17A [2]; the latter were identified as the most important source of IL-17A in the synovial fluid of PsA patients [3]. Memory CD4^+^ T cells that produce IL-17A share expression of C–C chemokine receptor 6 (CCR6) [4, 5]. Using flow cytometry, different CD4^+^CD45RO^+^ T helper cell subsets, including IL-17A producing cells, can be identified based on differential surface expression of chemokine receptors CD25, CCR6, CCR4, CXCR3, and CCR10: Th1 (CCR6^-^CD25^-/low^CCR4^-^CXCR3^+^), Th2 (CCR6^-^CD25^-/low^CCR4^+^CXCR3^-^), Th17 (CCR6^+^CD25^-/low^CCR4^+^CXCR3^-^CCR10^-^), Th17.1 (CCR6^+^CD25^-/low^ CCR4^-^CXCR3^+^CCR10^-^), Th22 (CCR6^+^CD25^-/low^ CCR4^+^CXCR3^-^CCR10^+^), double positive cells (DP; CCR6^+^CD25^-/low^CCR4^+^CXCR3^+^CCR10^-^), and double negative cells (DN; CCR6^+^CD25^-/low^CCR4^-^CXCR3^-^CCR10^-^), and regulatory T (Treg) cells (CD25^hi^) [4, 6, 7]. Treg cells can be further characterized by low expression of the surface marker CD127 [8].

In particular the CCR6^+^ memory Th cells are of interest for psoriasis and PsA. In other autoimmune diseases, such as rheumatoid arthritis (RA) and multiple sclerosis, differences in frequencies of CCR6^+^ memory Th cells were associated with a clinical phenotype or disease stage. Higher proportions of Th17.1, Th22, and DP cells were found in the peripheral blood of anti-citrullinated protein antibody positive (ACPA^+^) RA patients compared to ACPA^-^ patients [9]. In multiple sclerosis lower peripheral blood Th17.1 cell proportions during early disease were associated with rapidly progressive disease, while the cells accumulated in the cerebrospinal fluid of the patients [10, 11]. Although it is known that psoriasis and PsA patients have increased frequencies of IL-17A^+^ and IL-22^+^ CD4^+^ T cells in their peripheral blood and synovial fluid [3, 12], it is not clear if CCR6^+^ memory Th cells differ between psoriasis and PsA patients and if one or multiple subpopulations play a role in the transition of psoriasis to PsA.

Current knowledge on the identification and distinction of memory Th cell subsets comes from traditional/manual gating strategies based on visual inspection of cell populations using two-dimensional (2D) scatter plots [4, 12–15]. With the increased use of markers in current flow cytometry experiments, there is an exponential increase in the number of 2D scatter plots. This can result in missing relevant information, since traditional gating relies on the selection of defined cell populations based on specific markers. Unsupervised clustering through a machine learning algorithm, such as FlowSOM, provides information on how all markers are behaving on all cells and circumvents the risk of missing crucial information [16].

In the current study, we characterized the memory Th cell subsets in the peripheral blood of psoriasis patients with and without arthralgia, psoriasis patients that developed PsA in the following years and early PsA patients and compared these with sex and age-matched healthy volunteers using FlowSOM.

## Methods

### Patients

For this study, we included psoriasis patients from the Rotterdam Joint Skin cohort study. The Rotterdam Joint Skin cohort study collects data with the aim of early detection of PsA in the outpatient dermatology clinic. All newly diagnosed or newly referred psoriasis patients (≥ 18 years) were eligible to participate. Patients were recruited at the Maasstad Hospital in Rotterdam, The Netherlands. In addition, we included early PsA patients from the Dutch Southwest Early Psoriatic Arthritis cohoRt (DEPAR), of which the details are described elsewhere [17]. Briefly, patients with a new diagnosis of PsA (≥ 18 years) eligible to participate if they had not received systemic treatment with disease-modifying anti-rheumatic drugs (DMARDs) or biologicals for joint symptoms earlier. Healthy controls were recruited among Erasmus MC fellow employees. Written informed consent was obtained from all participants in both studies and healthy controls according to the Declaration of Helsinki. The Rotterdam Joint Skin study was approved by the Medical Research Ethics Committee United at the Maasstad Hospital, Rotterdam (2018-08). The DEPAR study was approved by the local Ethics Committee at Erasmus Medical Center, Rotterdam (2012-549). For the current study, we used available PBMCs from 22 patients in the Joint Skin study, 23 PsA patients in the DEPAR study and 17 sex and age-matched healthy controls.

### Data collection

Clinical data and blood samples were collected at baseline from psoriasis patients in the Rotterdam Joint Skin study. Clinical data on new joint complaints or PsA diagnosis was obtained from the medical records during the 2 years after baseline. A trained physician performed a full medical history and physical examination at baseline for psoriasis patients and collected data on medication use and clinical data, including swollen and tender joint count (resp. SJC 66 and TJC 68 joints), enthesitis at clinical examination (Leeds Enthesitis index, LEI) [18], dactylitis count, physician global Visual Analogue Scale (VAS), and psoriasis (Psoriasis Area and Severity Index, PASI) [19]. Regarding the symptom duration, patients of the Rotterdam Joint Skin study were asked how many years they experienced symptoms of their psoriasis, including nail disease, or how many years they experienced symptoms of PsA (in case of DEPAR). If a psoriasis patient had musculoskeletal symptoms at baseline, the patient was also evaluated by a rheumatologist. Ultrasound examination of the entheses was performed by the research physician for all psoriasis patients assessing the adapted MASEI protocol described earlier by Wervers et al. [20]. For the DEPAR study, trained research nurses collected the data on medication use and clinical data as described above.

### Flow cytometry staining

PBMCs were isolated from peripheral blood through ficoll and were stored in liquid nitrogen until use. Monoclonal antibody staining of PBMCs was performed as described earlier [9]. Briefly, cells were incubated for 15 min at room temperature with either the first panel including human antibodies: CCR10 (clone 314305; R&D), CD45RO (clone UCHL1; BD Biosciences), CCR6 (clone 11A9; BD Biosciences), CCR4 (clone 1G1; BD Biosciences), CD3 (clone UCHT1; BD Biosciences), cutaneous leukocyte antigen (CLA, clone HECA-452; BD Biosciences), CD4 (clone RPA-T4; BD Biosciences), and CXCR3 (clone G025H7; Sony), CD25 (clone bc96; Sony). CLA was added to the panel to visualize skin-homing T cells. Or cells were incubated with the second panel including CD3 (clone UCHT1; BD Biosciences), CD4 (clone SK3; BD Biosciences), CD25 (clone bc96; Sony), CD45RO (clone UCHL1; eBioscience), CD127 (clone hIL-7R-M21;BD Biosciences), and CLA (clone HECA-452; BD Biosciences). Subsequently, dead cells were excluded by incubation with Fixable Viability Dye eFluor506 (eBioscience) for 15 min at 4°C. All incubation steps were performed in the dark. Samples were measured on an LSRFortessa flow cytometer (BD Biosciences). Data were analyzed manually using FlowJo v10.7 software (Tree Star Inc. Ashland, OR). Additional file 1A and B depict the manual gating strategy of the two panels.

### FlowSOM-based unsupervised analysis and automated cell-type detection

Automated analysis of panel 1 was performed using the FlowSOM algorithm [16]. After exclusion of dead cells and doublets, CD3^+^CD4^+^CD45RO^+^CD25^-/low^ cells were gated. Data were compensated and transformed with an arcsinh transformation using cofactors calculated per channel using the FlowVS R package [21]. FCS files were downsampled to 6000 cells per sample resulting in a total of 3.72x10^5^ cells which were assigned to a self-organizing map with a 10x10 grid. Cells with a similar median expression of markers were grouped. A minimal spanning tree (MST) was built to visualize the clusters in branches. A final clustering of the data into metaclusters was performed using consensus hierarchical clustering as implemented in the ConsensusClusterPlus R package [22]. The number of meta-clusters was chosen using a delta-area plot as provided by ConsensusClusterPlus R package, which investigates the cluster number that provides the most stable clusters and best fits the data.

### Statistical analysis

Patient characteristics and baseline disease scores were described using simple descriptive analysis techniques. Comparisons between groups was performed using one-way ANOVA with a Tukey’s post hoc test. Pearson correlation test was used to calculate correlation coefficients and *p* values. *P* values were two-sided, and analyses were performed using STATA 14.0 and GraphPad Prism (version 9). *P*<0.05 was considered statistically significant.

## Results

### Patient characteristics

The baseline characteristics of the healthy controls, psoriasis patients, and PsA patients used in this study are demonstrated in Table 1. Twelve of the 22 psoriasis patients had no arthralgia at baseline, and two of these patients received a PsA diagnosis in the following 2 years. Seven psoriasis patients had arthralgia or enthesitis at baseline of which one patient received PsA diagnosis in the following 2 years. Unexpectedly, the three patients who developed PsA (subclinical PsA) had only a short symptom duration of 1 year. The three pre-clinical PsA patients also had a higher modified Madrid sonography enthesitis index (MASEI) score than the other psoriasis patients.

**Table 1:** Baseline characteristics of healthy controls and psoriasis and PsA patients

Characteristics	HC *n*=17	Psoriasis without arthralgia, *n*=12	Psoriasis with arthralgia, *n*=7	Subclinical PsA, *n*=3	PsA, *n*=23
Demographics					
Age, mean (SD)	41 ± 14	51.7 ± 10.6	40.4 ± 15.1	46 ± 11.8	41.3 ± 13.8
Male, *n* (%)	8 (47)	6 (50)	3 (43)	1 (33)	10 (43)
Symptom duration years, median (IQR)	-	12 (2–26)	15 (1–25)	1 (–)	1 (0.27–2.9)
Baseline disease scores					
PASI >0 (%)	-	9 (75)	7 (100)	3 (100)	18 (78)
Non-involved		2	0	0	5
Mild (>0 - ≤ 5)		8	5	2	13
Moderate/severe (>5)		1	2	1	5
TJC, median (IQR)	-	0	2 (1–4)	0 (0–2)	5 (2–8)
SJC, median (IQR)	-	0	0	0	2 (1–5)
Non-involved		10	6	3	1
Monoarthritis		0	0	0	8
Oligoarthritis		0	0	0	5
Polyarthritis		0	0	0	6
LEI > 0, *n* (%)	-	0	0	1	4
Global VAS, mean (SD)		27.5±9.8	28.9±13	35±21.2	19.7±17.1
Modified MASEI, median (IQR)	-	6.5 (4–10)	4 (1–14)	20.5 (3–22)	-
Power doppler signal in any enthesis, *n* (%)		2 (17)	0 (0)	1 (33)	-
Medication use					
DMARDs/fumaric acid, *n* (%)	-	1 (8.3)	1 (14.2)	0 (0)	1 (4.3)
Biological, *n* (%)		1 (8.3)	0 (0)	0 (0)	2 (8.7)

Data are shown as mean ± SD, *n* (%) or median (IQR). PASI score of 1 patient is missing, LEI score of 3 patients is missing, and MASEI score of 4 patients is missing. Abbreviations: *HC* healthy controls, *PsA* psoriatic arthritis, *PASI* Psoriasis Area Severity Index, *TJC* tender joint count, *SJC* swollen joint count, *LEI* Leeds Enthesitis Index, *MASEI* Madrid Sonography Enthesitis Index, *DMARDs* disease-modifying anti-rheumatic drugs

### Total frequencies of memory T helper cells and regulatory T cells do not differ between patients and healthy controls

**Fig. 1:**
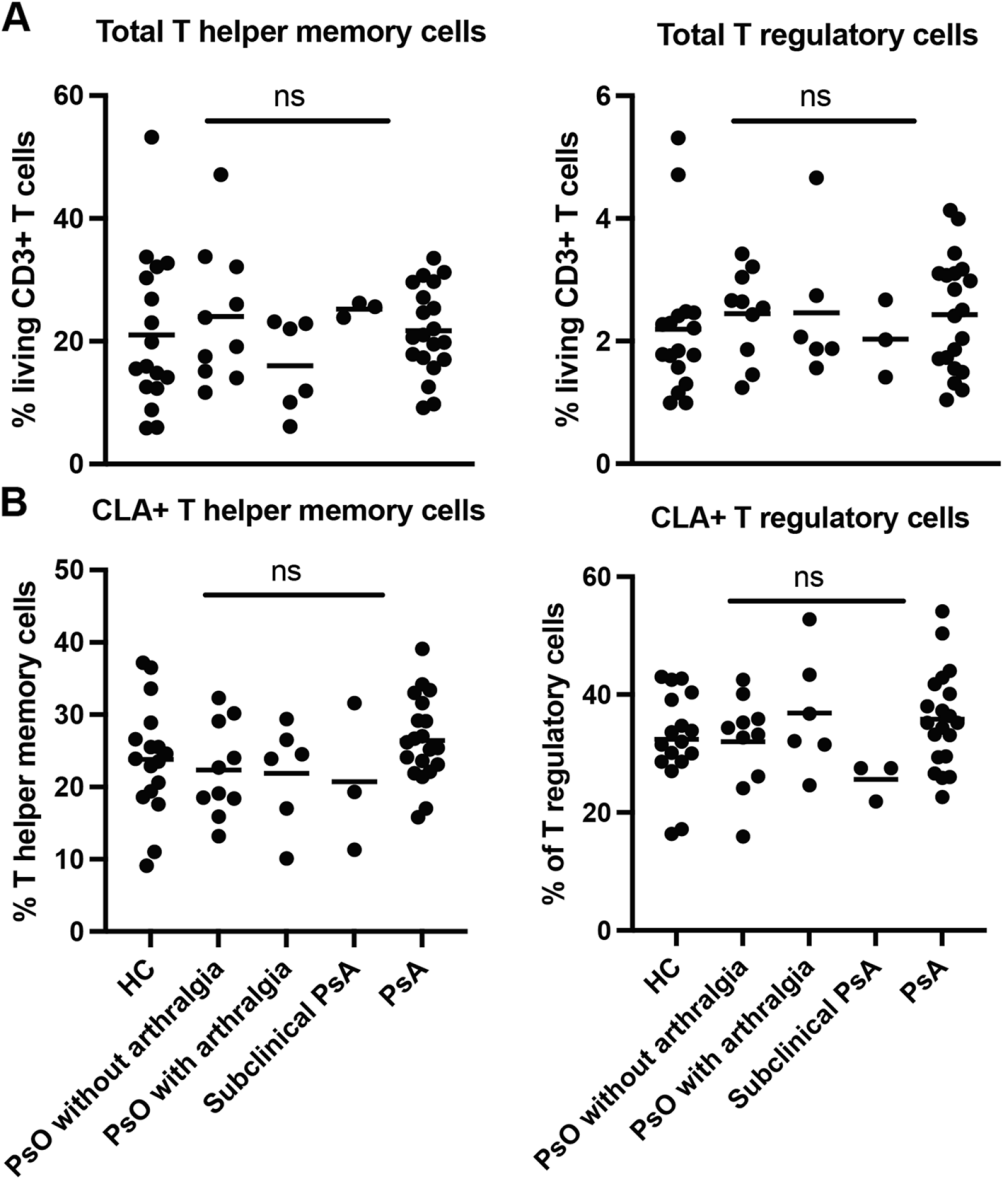
Manual gated percentages of total memory T helper and regulatory T cells. **A** Total percentage of memory T helper cells and regulatory T cells of total living CD3^+^ T cells. **B** Percentage of CLA^+^ cells of memory T helper cells or regulatory T cells. One-way ANOVA with Tukey post hoc test

Before using the machine learning algorithm FlowSOM, we manually gated the total frequency of memory Th cells and Treg cells to determine differences between patients and healthy controls. Fig. 1A shows that the percentages of memory Th cells and Treg cells of total CD3^+^ T cells does not differ between the groups. Fig. 1B also shows that all groups have similar percentages of CLA expressing memory Th and Treg cells.

### FlowSOM provides an in-depth overview of memory T helper cell distribution

**Fig. 2:**
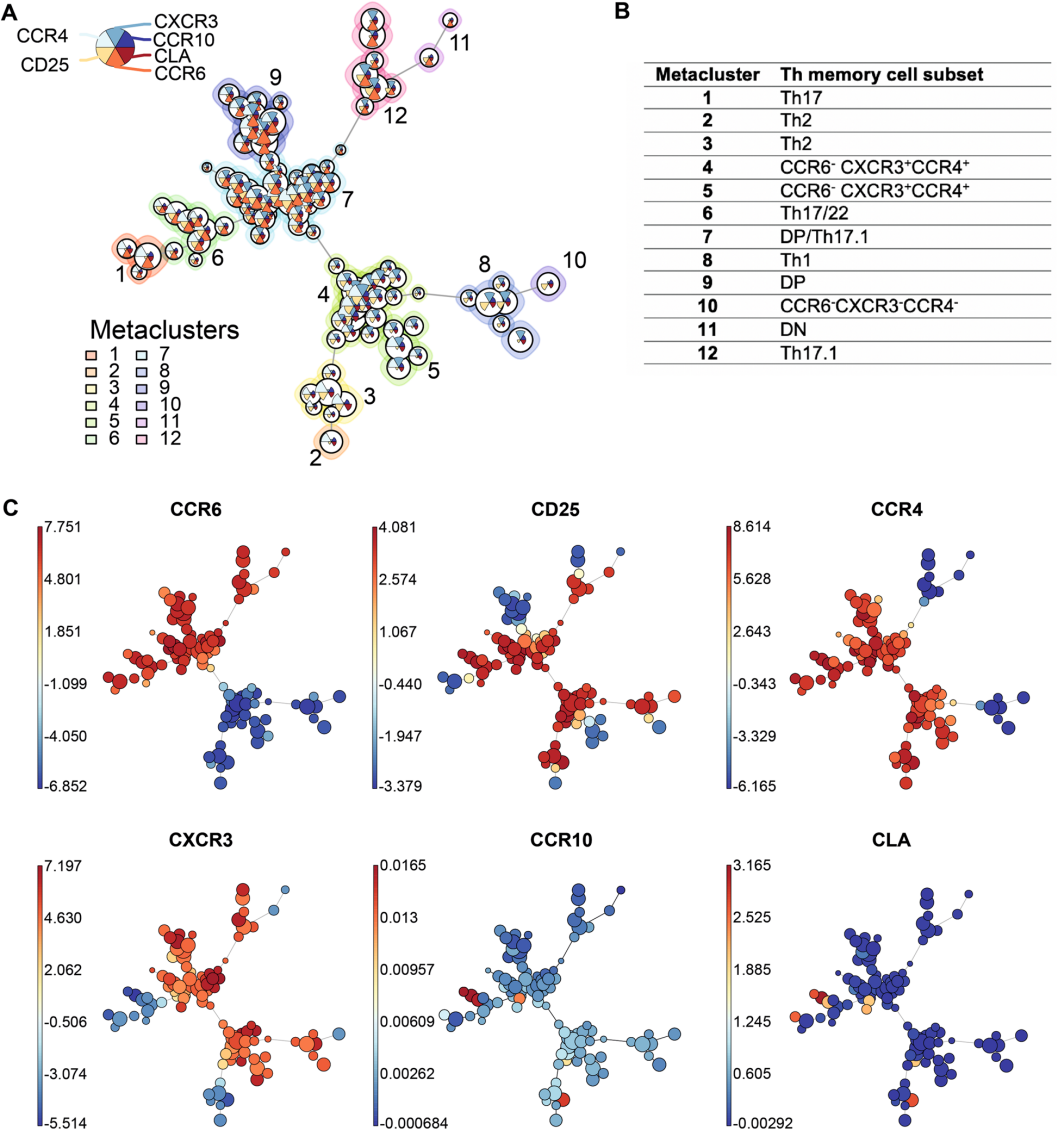
Unbiased clustering of memory T helper cells using FlowSOM algorithm. PBMCs of psoriasis and PsA patients and healthy controls were isolated and used for Flow cytometry. Doublets and dead cells were excluded. **A** Living CD3^+^CD4^+^CD45RO^+^CD25^low/int^ cells were gated for each subject and exported, arcsinh-transformed, down-sampled to 6000 cells per sample, and aggregated per group. The 3 aggregated files were used to generate a FlowSOM tree and cells were clustered into 100 nodes. The nodes were clustered in 12 metaclusters with hierarchical clustering, indicated by the background colors. **B** Table of metaclusters and the known labels for the different memory T helper subsets. **C** Median expression of the 6 individual surface markers (CCR6, CD25, CCR4, CXCR3, CCR10, and CLA) plotted in the generated FlowSOM tree

To further analyze memory Th cell subsets, we performed a FlowSOM analysis of pregated CD3^+^CD45RO^+^CD4^+^CD25^-/low^ cells, which were pooled from all samples (*n*=62; Fig. 2A). In addition to the chemokine receptors CCR6, CCR4, CXCR3, and CCR10, we also included CLA and CD25 in the FlowSOM analysis. FlowSOM and subsequent hierarchical clustering demonstrated 12 metaclusters based on the used markers. FlowSOM identified all previously identified memory Th cell subsets (Fig. 2B). The FlowSOM tree clearly discriminated between CCR6^+^ and CCR6^-^ memory Th cells (Fig. 2C). CCR6^+^CCR4^+^CXCR3^-^ Th17 cells are identified in metaclusters 1 and 6. A small cluster of Th17 cells also expresses CLA, the skin-homing receptor. Metacluster 6 also includes the Th22 cells (CCR6^+^CCR4^+^CXCR3^-^CCR10^+^ cells). All Th22 cells express CLA. Metaclusters 1 and 6 mostly differ in their CD25 expression (Fig. 2C). Metacluster 12 relates to the Th17.1 cells, which express CCR6, CXCR3, but not CCR4 or CCR10 (Fig. 2C). These cells do not express CLA. The DP cells are found in the largest metaclusters 7 and 9 and the DN cells in the smallest metacluster 11. Within metacluster 7 (DP cells), some small clusters of cells express CCR10 and CLA. FlowSOM also depicts the diversity in CCR6^-^ memory Th subpopulations with metacluster 8 (Th1 cells) and metaclusters 2 and 3 (Th2 cells), but also previously unidentified metaclusters 4 and 5 with both CCR4 and CXCR3 expression and metacluster 10, which does not express both chemokine receptors (Fig. 2C).

### FlowSOM analysis is more accurate in discriminating memory Th cell subsets than manual gating

**Fig. 3:**
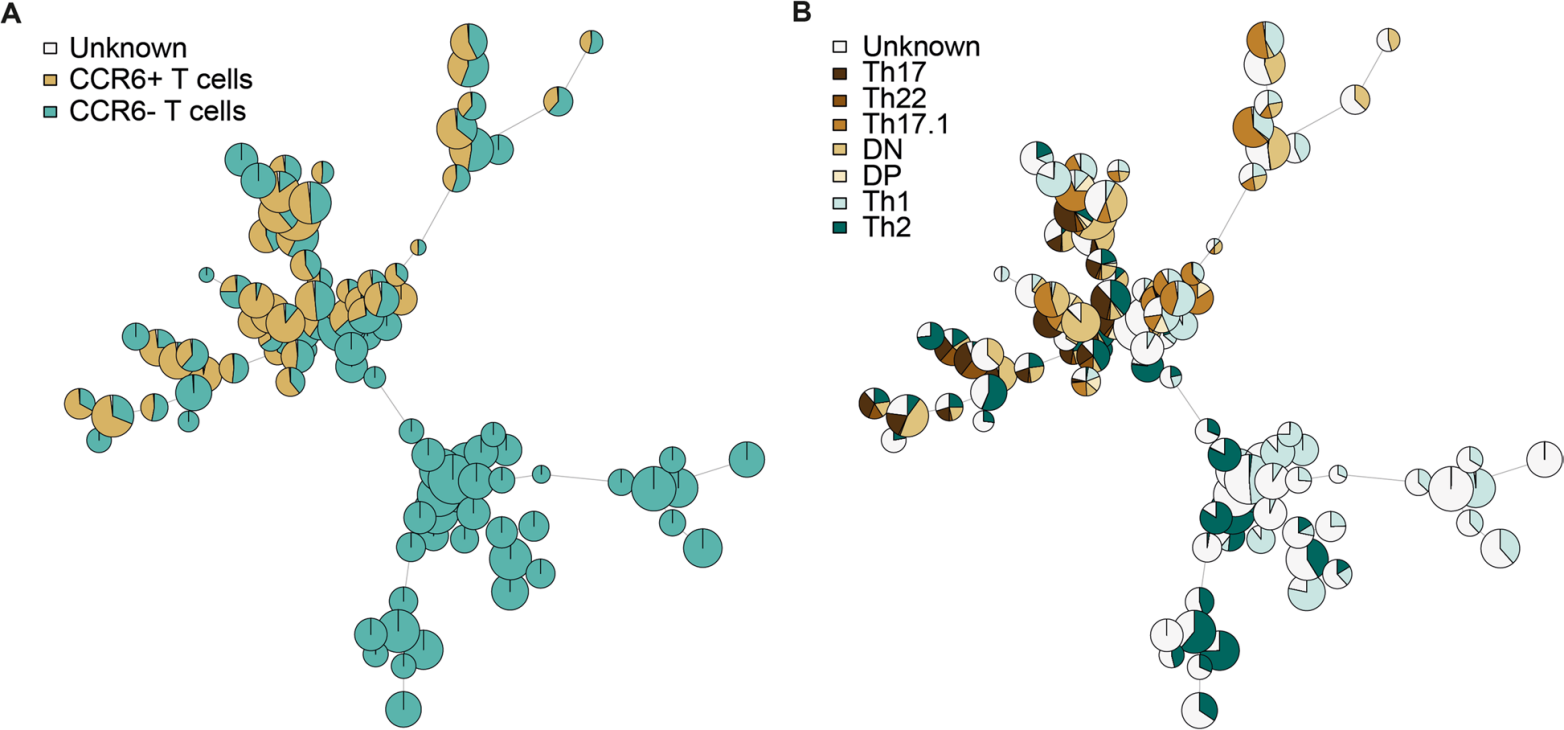
Manual gating overlay of FlowSOM tree for CCR6^+^ and CCR6^-^ Th cell subpopulations. PBMCs of psoriasis and PsA patients and healthy controls were isolated and used for Flow cytometry. Doublets and dead cells were excluded. Cells were analyzed as described in Fig. 1. **A** Cells that were gated manually as CCR6^+^ or CCR6^-^ are indicated with yellow or green on the FlowSOM tree. **B** Cells that were gated manually as CCR6 subpopulations (Th17, Th22, Th17.1, DP, DN, or Th1 and Th2) are indicated by colors on the FlowSOM tree

Next, we compared the results of FlowSOM analysis with manual gating of the memory Th cells by making an overlay of the results of FlowSOM versus manual gating analysis. The manual gating strategy is shown in Additional file 1B. Using the overlay, we found that by manually gating the cells, some CCR6^+^ Th cells were wrongly gated as CCR6^-^ (Fig. 3A). Figure 3B demonstrates differences in the gating of the CCR6^+^ and CCR6^-^ Th cell subsets between manual gating and FlowSOM.

### Psoriasis and PsA patients have a different memory T helper cell distribution compared to healthy controls

**Fig. 4:**
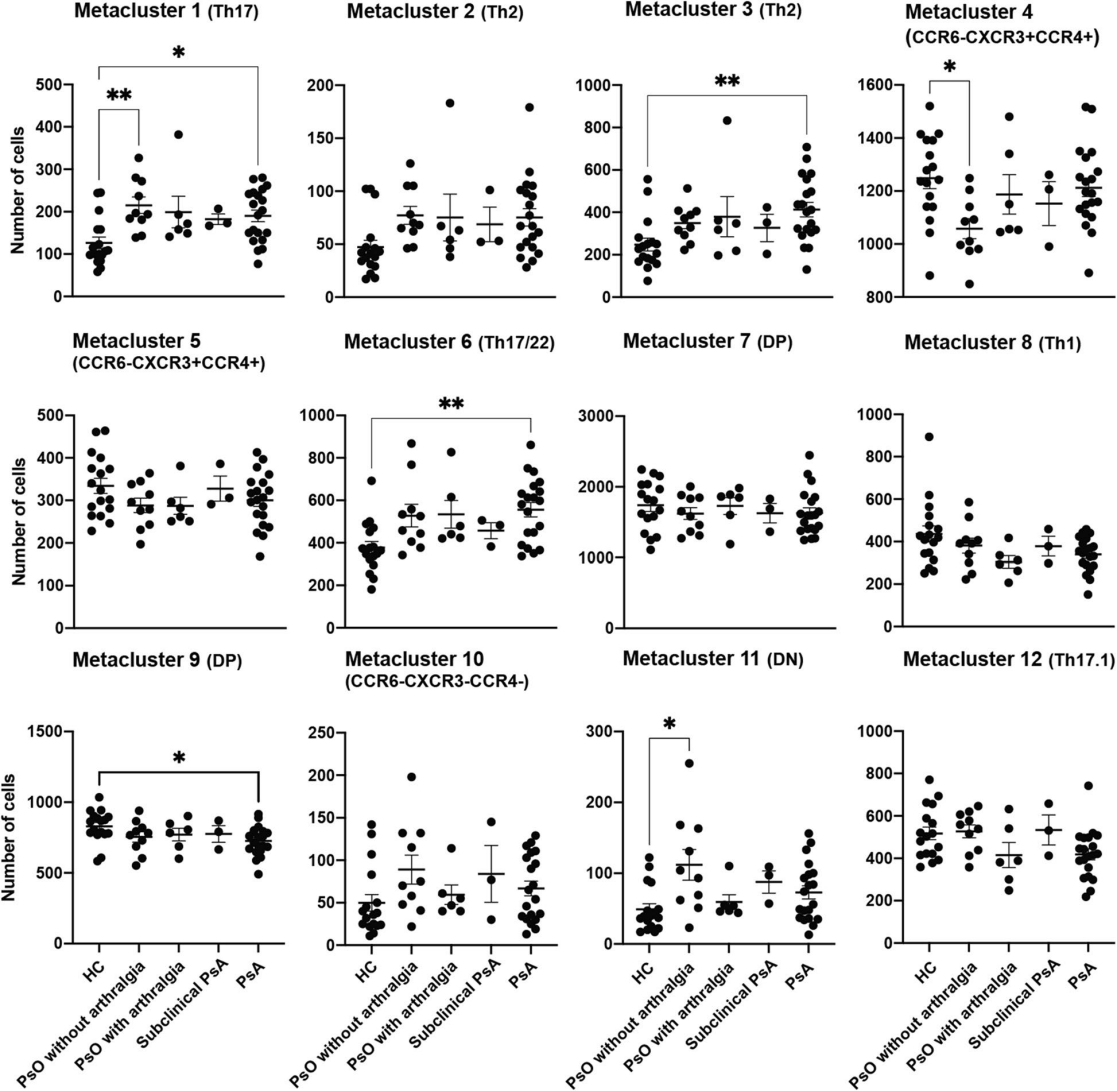
Differences between memory CD4^+^ T cells of PsA, PsO, and HC analyzed with FlowSOM. PBMCs of psoriasis and PsA patients and healthy controls were isolated and used for Flow cytometry. Doublets and dead cells were excluded. Cells were analyzed as described in Fig. 1. Scatter dot plots of the total number of cells in the metaclusters identified by FlowSOM are depicted as mean ± SEM. One-way ANOVA with Tukey’s post hoc test. *p<0.05 and **p<0.01

Subsequently, we compared the number of cells in each identified metacluster between psoriasis patients with or without arthralgia, psoriasis patients who developed PsA during follow-up (subclinical PsA), early PsA patients and healthy controls (Fig. 4). Although the Th cell profile of patients using systemic medication was not different from those who did not use systemic medication, they were excluded in this analysis (Figure S2). The number of cells in metaclusters 1 (Th17 cells), 6 (Th17/22 cells), and 3 (Th2 cells) was significantly higher in PsA patients compared to HC, while the number of cells in metacluster 9 (DP cells) was significantly lower compared to HC. Metacluster 4 (CCR6^-^ DP cells) was decreased and metaclusters 1 (Th17), and 11 (DN cells) were increased in psoriasis patients without arthralgia compared to HC. We did not find any significant differences in any of the identified metaclusters between the PsO (with and without arthralgia), sub-clinical PsA and PsA patients.

### Skin disease severity and the number of tender joints correlate with Th17 and Th17.1 cells

**Fig. 5:**
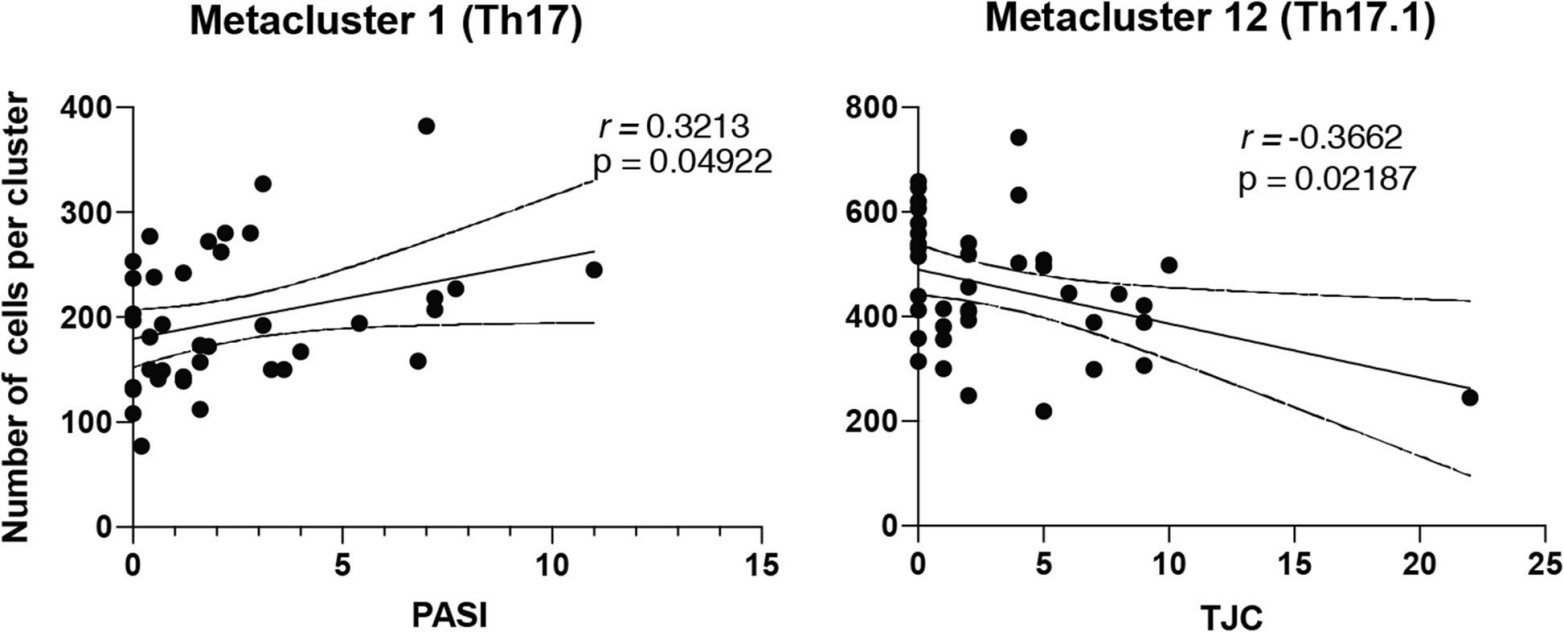
Significant correlations between metacluster 1 (Th17 cells) and PASI score and metacluster 12 (Th17.1 cells) and TJC. PBMCs of psoriasis and PsA patients were isolated and used for flow cytometry. Doublets and dead cells were excluded. Cells were analyzed as described in Fig. 1. Pearson correlation coefficient

Next, we assessed whether the frequency of the identified clusters correlated with disease severity measures (PASI, LEI, MASEI, TJC, SJC; Fig. 5) in PsA and psoriasis patients. Among psoriasis and PsA patients, the PASI score for psoriatic skin involvement was positively correlated with Th17 (metacluster 1) cell numbers (*r*= 0.32, *p*<0.05). The number of tender joints were negatively correlated with Th17.1 cells (metacluster 12; *r*=−0.37, *p*<0.05). We found no significant correlations between manually gated Th subpopulations and disease severity measures.

## Discussion

In this study, we identified cell clusters of memory Th cells based on the markers CCR6, CCR4, CXCR3, CCR10, CLA, and CD25 by using an unsupervised machine learning algorithm FlowSOM. In addition, the role of these clusters in the transition from psoriasis to PsA was investigated. Psoriasis and PsA patients have a different memory Th cell distribution compared to healthy controls. However, specific memory T helper subpopulations only present in subclinical PsA patients or psoriasis patients with arthralgia were not identified. Also, there was no population with significantly higher or lower numbers present in these patients. However, we did find significant correlations between Th17 cell numbers and PASI score and Th17.1 cell numbers and TJC in PsA and psoriasis patients.

Psoriasis precedes arthritis in most PsA patients by an average of 7 years [23], which provides an opportunity for early detection of PsA if we can identify those psoriasis patients at risk. Since a majority of PsA patients have progressive joint damage, which is even worse if the diagnosis and start of treatment is delayed [24], it is important to advance our knowledge on the transition of psoriasis to PsA. In our study, we divided our psoriasis patients into 3 groups: psoriasis patients without arthralgia, psoriasis patients with arthralgia, which are proposed to be at risk for developing PsA, and psoriasis patients who developed PsA during 2-year follow-up (subclinical PsA). Despite our short follow-up time, we were able to identify 3 patients with subclinical PsA. Although we could analyze only 3 patients, it is important to describe our current results, because the number of studies investigating immunological mechanisms in psoriasis patients developing PsA is limited.

All proposed models for psoriasis to PsA transition identify aberrant activation of the IL-17-IL-23 axis as an important factor in the transition. We used the advanced machine learning algorithm FlowSOM to analyze memory Th cell subsets, including Th17 cells, to investigate if there are differences between psoriasis and PsA patients. FlowSOM is a fast and highly accurate algorithm for cell population classification and discovery [16, 25]. In our study, the FlowSOM algorithm identified all previously known memory Th cell subsets, including Th1, Th2, Th17, Th22, and Th17.1 based on surface expression of the chemokine receptors and CD25 and CLA. In line with earlier findings of increased frequencies of IL-17A and IL-22 producing cells in psoriasis and PsA [3, 12], we also found increased numbers of Th17 cells (metaclusters 1) in both psoriasis and PsA patients compared to healthy controls. Psoriasis patients without arthralgia showed lower numbers of CCR6^-^CXCR3^+^CCR4^+^ cells and higher numbers of CCR6^+^ DN cells. T cells isolated from psoriatic skin lesions are known to express CXCR3 and CCR4, which could explain that CCR6^-^ CXCR3^+^CCR4^+^ cells migrate to the skin and are lower in the peripheral blood [26]. The origin of CCR6^+^ DN cells, which also applies to CCR6^+^ DP cells, is unclear and they might represent transitional or intermediate T cells from the more stable Th17 or Th17.1 cells [5]. In RA, both CCR6^+^ DN and DP were able to strongly activate synovial fibroblasts as opposed to CCR6^-^ memory Th cells [27]. Early PsA patients had significantly increased number of cells in metacluster 3 (Th2 cells) and Th17/22 cells (metaclusters 1 and 6), and lower numbers of CCR6^+^ DP cells (metacluster 9). Metacluster 3 included Th2 cells that also co-express the skin-homing receptor CLA and CCR4, suggesting that these cells might increasingly recirculate between the skin and blood in early PsA patients. Lower numbers of CCR6^+^ DP cells could indicate that these cells migrate more to the joints, entheses or skin.

We did not identify any specific metacluster as significantly different in subclinical PsA patients compared to HC. The low number of patients used in our analysis could be a potential explanation for this, since PsA patients had differences in metaclusters 1, 6, and 9 compared to HC.

Th17 cells (metacluster 1) positively correlated with PASI scores, and Th17.1 cells (metacluster 12) negatively correlated with tender joints. These results suggest a role for Th17 and Th17.1 subpopulations in the pathogenesis of both psoriasis and PsA and support a role for these cells especially in psoriatic skin lesions. In autoimmune diseases, such as multiple sclerosis, RA, and Crohn’s disease, Th17.1 cells were decreased in the peripheral blood and migrated to the inflamed tissues [5, 11]. However, we could not find lower numbers of Th17.1 cells in our subclinical PsA patients. Future research including more subclinical PsA patients might provide a clearer view on the role of Th17.1 cells and other CCR6^+^ Th subpopulations.

FlowSOM is also useful to identify smaller populations of cells which are difficult to visualize with manual gating. For instance, we detected a small cluster of CLA^+^ Th17 cells (metacluster 1), which did not co-express CCR10, which is also a skin-homing receptor, and a small cluster of CLA^+^ Th17 cells, which co-expressed CCR10 (metacluster 6). In addition to new clusters of CLA^+^ Th cells, FlowSOM also visualized differences in CD25 expression between clusters. CD25, also known as the interleukin-2 receptor alpha chain, is important for T cell proliferation and activation. While we manually excluded Th cells that express high levels of CD25, FlowSOM analysis did distinguish between moderate and low expression of CD25. In our analysis, metaclusters 1 (Th17 cells) and 6 (Th17/22 cells) differed in their CD25 expression, with metacluster 6 consisting of CD25 low expressing cells and metacluster 1 consisting of CD25^-^ cells. This might indicate that metacluster 6 includes more activated Th17/22 cells.

Our study has several strengths and limitations. One of the strengths of our study is that we included a sufficient amount of 45 psoriasis and PsA patients and 17 healthy controls, all sex and age-matched, to provide insight in the distribution of CCR6^+^ Th subpopulations among psoriasis and PsA patients. Unfortunately, we only had three patients who had converted to PsA during a follow-up period of 2 years. Identifying psoriasis patients that convert to PsA is a major challenge in the PsA field. To overcome this challenge, the Rotterdam Joint Skin study not only includes newly diagnosed psoriasis patients, but also psoriasis patients who are newly referred to a dermatologist from the general practitioner. Another strength of our study is the use of a computational flow cytometry strategy with an objective and unbiased machine-learning algorithm for the analysis, which enabled us to gain a full overview of the Th cell clusters. Our study clearly demonstrates that analysis of CCR6^+^ subpopulations by manual gating deviates to a certain extent from the unbiased FlowSOM analysis. This deviation is larger for markers which have lower expression on cells. Since FlowSOM provides the opportunity to identify all possible combinations of markers, it can be used for identification of biomarkers in diseases instead of manual gating.

## Conclusions

In summary, our results demonstrate that both psoriasis and PsA patients have an increased number of circulating Th17 and Th22 cells compared to healthy controls. In addition, the circulating memory Th cell populations differ between psoriasis and PsA patients with cell type specific correlations with clinical scores. However, we did not find differences in circulating CCR6^+^ Th subpopulations between psoriasis patients with or without arthralgia or psoriasis patients who developed PsA in the following 2 years. Future research on the transition from psoriasis to PsA may focus more on tissue-specific changes in psoriatic skin lesions and investigate other circulating cell types that might play a role herein.

## Supplementary Information

**Additional file 1.** The manual gating strategy.

## Data Availability

The datasets used and/or analyzed during the current study are available from the corresponding author on reasonable request.

## Abbreviations

DMARDs: Disease-modifying anti-rheumatic drugs
CCR: C–C chemokine receptor
CLA: Cutaneous leukocyte antigen
CXCR: C–X–C chemokine receptor
DEPAR: Dutch Southwest Early Psoriatic Arthritis cohoRt
DN: Double negative
DP: Double positive
HC: Healthy controls
IL: Interleukin
LEI: Leeds enthesitis index
MASEI: Madrid Sonography Enthesitis Index
PASI: Psoriasis Area Severity Index
PBMC: Peripheral blood mononuclear cells
PsA: Psoriatic arthritis
RA: Rheumatoid arthritis
SJC: Swollen joint count
SOM: Self-organizing maps
Th: T helper
TJC: Tender joint count
Treg: Regulatory T cell
