# Supplementary figures and images for: Characterizing memory T helper cells in patients with psoriasis, subclinical, or early psoriatic arthritis using a machine learning algorithm
Source: Arthritis Res Ther. 2022 Jan 19;24:28. doi: 10.1186/s13075-021-02714-5 (PMC8767727; doi:10.1186/s13075-021-02714-5)

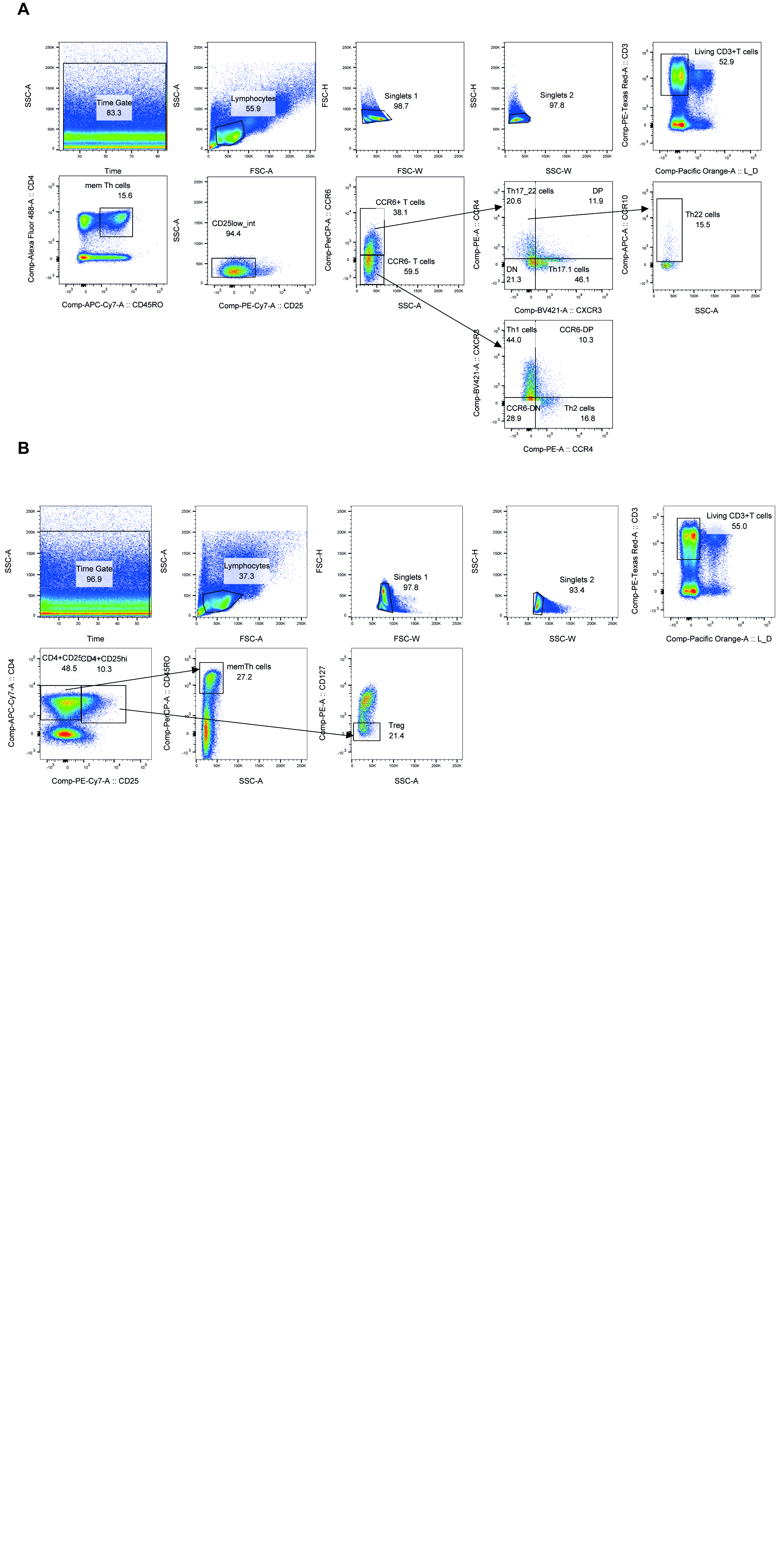

Supplement: Supplementary file 1 — Additional file 1. The manual gating strategy. [file 13075_2021_2714_MOESM1_ESM.tif]
